# Amplicon microbiome sequencing of compost from conventional and redesigned compost buckets

**DOI:** 10.1128/mra.00808-24

**Published:** 2025-02-25

**Authors:** Elizabeth Klosko, Elizabeth Hutchison, Ahmad Almomani

**Affiliations:** 1 Chemistry Department, State University of New York, Geneseo, New York, USA; 2 Biology Department, State University of New York, Geneseo, New York, USA; 3 Mathematics Department, State University of New York, Geneseo, New York, USA; University of Maryland School of Medicine, Baltimore, Maryland, USA

**Keywords:** compost, microbiome, 16S rRNA

## Abstract

Here, we report using amplicon sequencing to assess microbial growth in both conventional and pyramid-shaped compost buckets. Proteobacteria and Firmicutes were the primary bacterial phyla present, and Ascomycota the primary fungal phylum present. Bucket shape did not significantly affect microbial community composition.

## ANNOUNCEMENT

Composting is the controlled decomposition of organic wastes into nutrient-rich biological metabolites, and it has many environmental benefits (1). One barrier to increasing composting use is compost odor (2). Microbes are integral to composting and produce waste products that contribute to compost odors (3). Previous studies indicated potentially reduced microbial growth on compost if contained in a pyramid shape (4, 5). We designed a new, pyramid-shaped compost bucket for the SUNY Geneseo campus (14454, NY). DNA extracted from compost was used to assess microbial composition in conventional and pyramid-shaped buckets.

Poll data from campus compost bucket users informed our ingredient ratios: banana (45.8%), stewed vegetables (28.5%), tomato (23.1%), compostable take-out container (1.93%), and napkin (0.67%). Compost was blended with sterile water (1:1 wt/vol) after 4 weeks and flash frozen at −80°C in 0.5 g aliquots. Bead beating (1 min, 0.25 g of 0.5 mm glass beads) using a mini bead beater (BioSpec) and 10 min of vortexing was performed prior to extraction. DNA was extracted using a Qiagen DNeasy PowerSoil Kit according to the manufacturer’s protocol. Three extractions were pooled for each sample to maximize DNA yield. Three samples were extracted for each bucket type and sent to SeqCenter (Pittsburgh, PA) for 16S rRNA and ITS sequencing. The entire experiment was repeated once for an additional biological replicate.

SeqCenter prepared samples using Zymo Research’s Quick-16S kit with primers targeting the 16S gene V3/V4 regions for bacterial sequencing (forward: CCTACGGGDGGCWGCAG, CCTAYGGGGYGCWGCAG; reverse: GACTACHVGGGTATCTAATCC, GACTACNVGGGTMTCTAATCC). Fungal samples were prepared using Zymo Research’s Quick-ITS kit with primers targeting the ITS2 region (forward: GCATCGATGAAGAACGCAGC; reverse: TCCTCCGCTTATTGATATGC). After clean-up and normalization, samples were sequenced on a P1 600 cycle NextSeq2000 flowcell to generate 2 × 301 bp paired-end reads, and outputs are summarized in Table 1. For 16S rRNA data, primers were removed using cutadapt (v.4.4) (6), and analyzed using the pipeline described in Lee ( 7). DADA2 (v.1.32.0) (8) was used to generate amplicon sequence variants (ASVs) and taxonomy assigned using the SILVA v138 database (9). Data were imported into phyloseq (v.1.48.0) (10) for additional analysis, and DeSeq2 (v.1.44.0) was used to test for differences in ASV abundance (11). The pipeline was carried out in R (v.4.4.0) (12), and ggplot2 (v. 3.5.1) (13) was used for data visualization. Fungal ITS analysis was performed via SeqCenter using bcl-convert (v.4.1.5) and Qiime2 (v.2021.11) (14). For all software, default parameters were used unless otherwise noted.

**TABLE 1:** Summary of sequencing read outputs

Sequencing type	Sample	Total read pairs	% Basepairs > Q30	Average read lengths (bp)
Bacterial	1O	322,697	88.3	301
	2O	358,503	88.6	301
	3O	342,570	89	301
	4O	192,907	92.6	301
	5O	222,850	92.6	301
	6O	197,928	93	301
	1P	274,715	87.5	301
	2P	363,238	89.3	301
	3P	316,438	88.8	301
	4P	119,773	93.1	301
	5P	139,270	88.6	301
	6P	204,874	93	301
Fungal	1O	249,971	88.4	301
	5O	86,781	88.9	301
	2P	272,943	89.1	301
	5P	116,380	88.6	301

**Fig 1:**
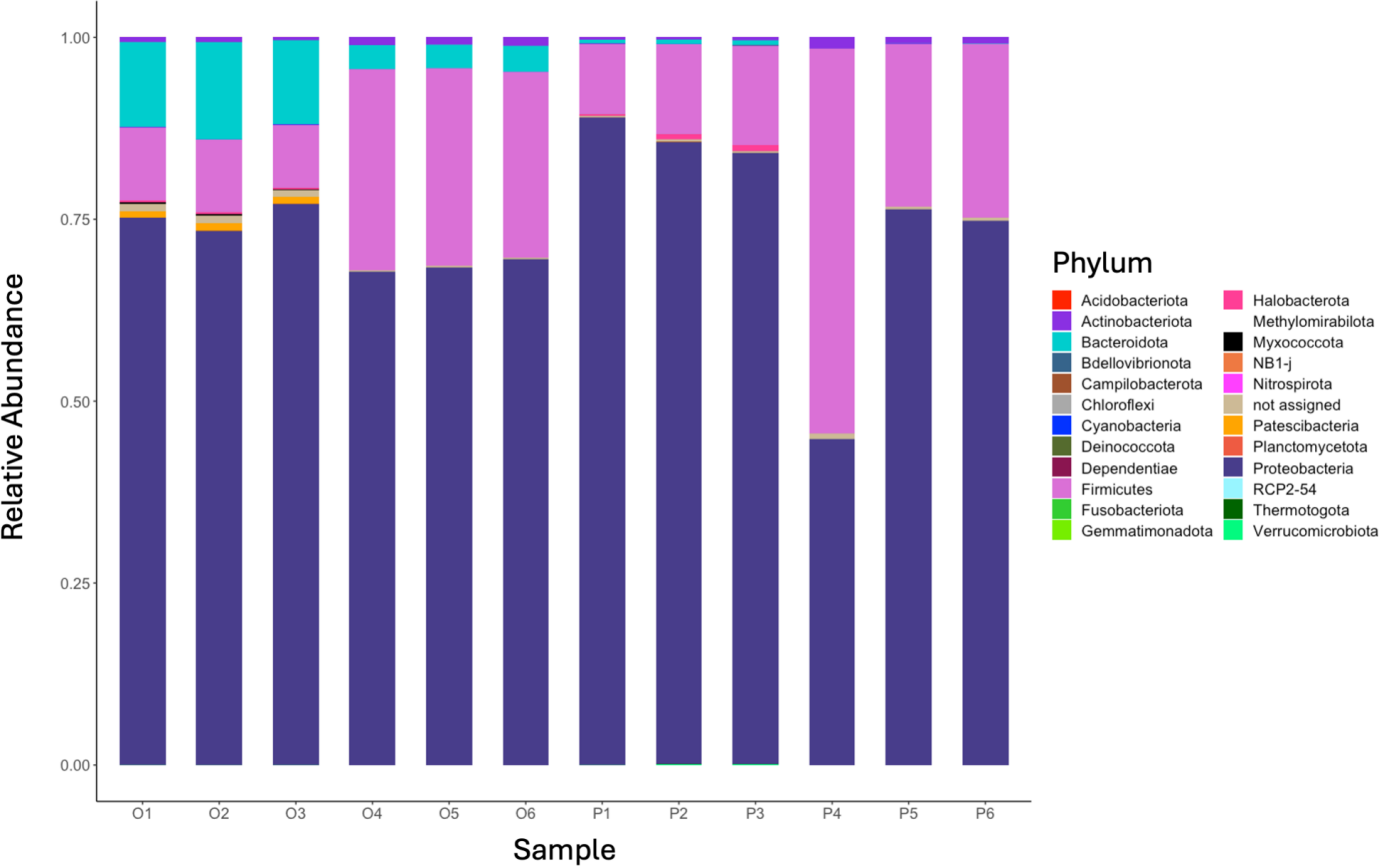
Relative ASV abundance in original (O) and pyramid (P) shaped compost buckets. Samples 1–3 are from the first biological replicate, and samples 4–6 are from the second biological replicate.

Major bacterial phyla observed were Proteobacteria and Firmicutes (Fig. 1), and the major fungal phylum observed was Ascomycota. Results are consistent with previous observations of compost microbiomes (15, 16), and significant differences were not observed between bucket types.

## Data Availability

Data including bacterial 16S rRNA and fungal ITS reads (SRA SRP512203) are available via NCBI’s BioProject accession number PRJNA1120438.
